# Mapping of pituitary stress-induced gene regulation connects Nrcam to negative emotions

**DOI:** 10.1016/j.isci.2022.104953

**Published:** 2022-08-17

**Authors:** Maria Belland Olsen, Ann-Christin Sannes, Kuan Yang, Morten Birkeland Nielsen, Ståle Valvatne Einarsen, Jan Olav Christensen, Ståle Pallesen, Magnar Bjørås, Johannes Gjerstad

**Affiliations:** 1 Research Institute of Internal Medicine, Oslo University Hospital, Oslo, Norway; 2 Faculty of Medicine, University of Oslo, Oslo, Norway; 3 National Institute of Occupational Health, Oslo, Norway; 4 Department of Psychosocial Science, University of Bergen, Bergen, Norway; 5 Department of Microbiology, Oslo University Hospital, Oslo, Norway; 6 Department of Clinical and Molecular Medicine, Norwegian University of Science and Technology, Trondheim, Norway

**Keywords:** Biological sciences, Neuroscience, Clinical neuroscience, Endocrinology

## Abstract

Environmental stressors such as repeated social defeat may initiate powerful activation of subconscious parts of the brain. Here, we examine the consequences of such stress (induced by resident-intruder paradigm) on the pituitary gland. In male stressed vs. control rats, by RNA- and bisulfite DNA sequencing, we found regulation of genes involved in neuron morphogenesis and communication. Among these, *Neuronal cell adhesion molecule* (*Nrcam*) showed reduced transcription and reduced DNA methylation in a region corresponding to intron 1 in human *NRCAM*. Also, genetic variability in this area was associated with altered stress response in male humans exposed to repeated social defeat in the form of abusive supervision. Thus, our data show that the pituitary gene expression may be affected by social stress and that genetic variability in *NRCAM* intron 1 region influences stress-induced negative emotions. We hope our shared datasets will facilitate further exploration of the motions triggered by social stressors.

## Highlights

• Social stress-induced pituitary gene regulation was characterized in rats
• Here, genes involved in neuron morphogenesis and communication were regulated
• Both expression and methylation of the *Nrcam* gene were affected
• Genetic variability in *NRCAM* in humans influenced stress-induced negative emotions

## Graphical abstract

**Figure:**
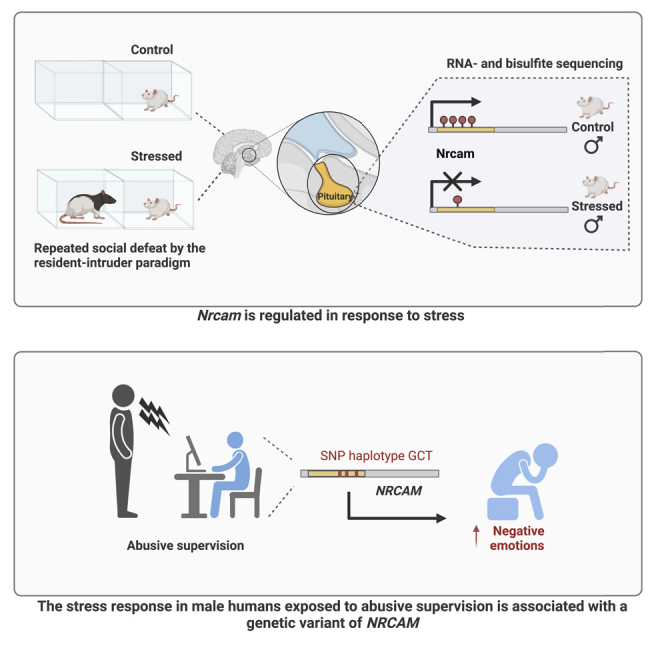

## Introduction

Previous findings suggest that environmental stressors, such as repeated social defeat (Jacobsen et al., 2020; Rajalingam et al., 2020), may be associated with fear and behavioral alterations (McKim et al., 2018; Wohleb et al., 2014). For example, repeated social defeat in social mammals triggers activation of subconscious parts of the brain, which in turn promotes nervousness (Rajalingam et al., 2021; Wohleb et al., 2014). In rats, exposure to social stress may be seen as momentary submissive supine posture, freeze or flight, and later vulnerability toward cued fear memory dysfunction (Mancini et al., 2021). In humans, the changes may be manifested as “negative affect”, i.e. being afraid, nervous, upset, hostile, or ashamed (Thompson, 2007).

We have recently demonstrated that repeated social defeat (the resident-intruder paradigm) is a strong stressor that may induce behavioral changes associated with reduced weight gain and increased impulsivity or locomotion 24 h after the last experience of social defeat in rats (Jacobsen et al., 2020; Rajalingam et al., 2020). A correlation between reduced social behavior and increased plasma (nor)epinephrine in animals exposed to social stress has also been observed (Rajalingam et al., 2021). Thus, it seems plausible that such social stressors, i.e., the asymmetry of power (Rajalingam et al., 2020), may trigger long-term changes in the hypothalamus-pituitary-adrenal (HPA) axis and subsequent stress-related biological processes underlying fear and nervousness (Smith and Vale, 2006).

At the cellular level in mice, repeated social defeat may lead to changes in both mRNA expression levels (Bagot et al., 2015; Bondar et al., 2018) and DNA methylation patterns (Hing et al., 2018) in several brain areas, including the hippocampus and medial prefrontal cortex (mPFC). Also, earlier data show that repeated social defeat triggers downregulation of genes involved in axonal guidance, cell adhesion, and neuroplasticity—in the mPFC—which in turn may induce a depressive-like state (Bondar et al., 2018; Pizarro et al., 2004). This indicates that complex neuronal biological processes in rodents may be activated by such strong social stressors.

In addition, several lines of evidence from human studies show that psychosocial stressors increase the risk of mental distress (Huang et al., 2019; Mitchell and Ambrose, 2007; Nielsen et al., 2017) and a variety of health complaints (Glambek et al., 2018; Rajalingam et al., 2019; Sannes et al., 2021). Thus, persistent stress exposure, in particular where the power imbalance is an essential aspect (Tepper, 2000), may support anxiety and negative affect (Vie et al., 2012). One important stressor that may affect mental health is exposure to abusive supervision, i.e., non-physical hostile behaviors, such as uncontrolled outbursts, inappropriate blaming, and public ridicule, from supervisors directed at subordinates (Huang et al., 2019; Tepper, 2000). As for specific mental health problems, exposure to abusive supervision has been linked to symptoms of mental distress, including depression and self-regulation impairment, for review see (Tepper et al., 2017).

Stress-induced behavioral responses may be associated with activation of the limbic system, the periaqueductal gray, and the hypothalamus (Harper et al., 2018; Herman et al., 2005). Furthermore, evidence exists that social stress in rodents, as well as in humans, may affect gene expression and methylation of hippocampus (Zhang et al., 2013). Therefore, such changes in the pituitary triggered by strong social stressors like abusive supervision seem also likely. However, hypothesis-free research addressing the association between social stress, pituitary (epi)genetics and emotions, is lacking. Hence, the effect of social stress on pituitary gene expression or methylation—and the possible link between social stress and negative affect—was explored in the present paper.

First, pituitary tissue from stressed rats (resident-intruder stress paradigm) was analyzed to determine global pituitary RNA expression and pituitary genomic levels of DNA methylation, i.e., 5- methylcytosine (5mC). Next, the association between abusive supervision, genetics, and negative affect was examined with special focus on genetic variability. The aim of the study was to provide new knowledge about how exposure to social stress, through genetic mechanisms, may lead to long-term changes in the HPA axis and negative affect.

## Results

### Repeated social defeat alters pituitary gene expression

**Figure 1:**
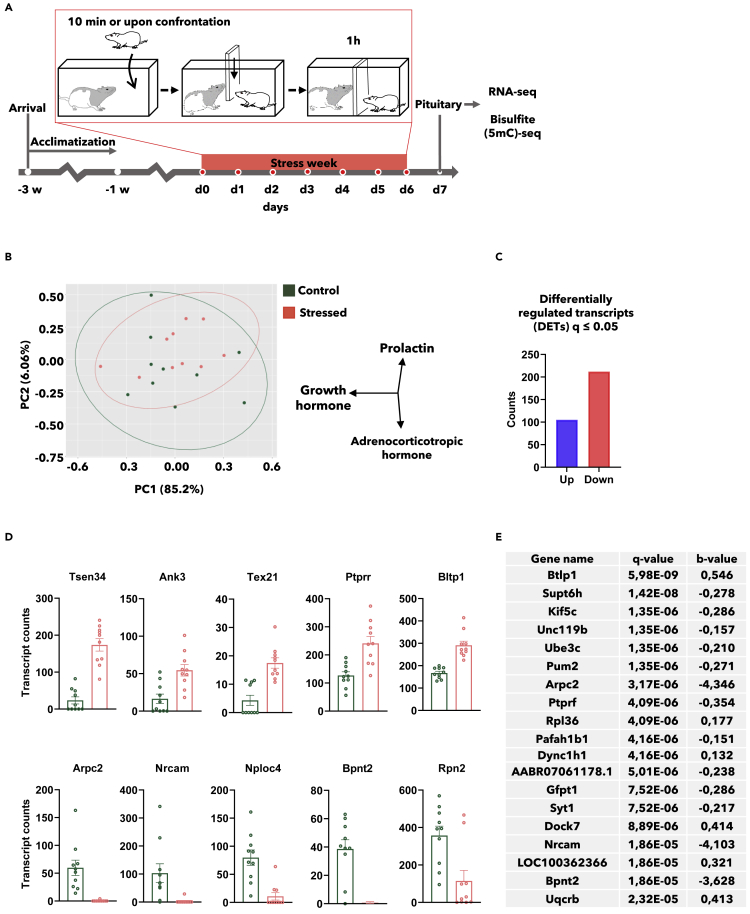
Repeated social defeat alters pituitary gene expression (A) Experimental set-up of the resident-intruder paradigm; male Sprague Dawley test rats were exposed to repeated social stress by dominant Long Evans rats for 1 h each day for seven consecutive days (n = 10). Sprague Dawley test rats not exposed to stress were used as controls (n = 10). Pituitary tissue was harvested 24 h after last stress exposure. (B) Principal component analysis (PCA)-plot including all detected genes by RNA sequencing in pituitary tissue harvested after 7 days of daily social defeat. The main driver of variance was growth hormone (ENSRNOT00000015818.5), followed by prolactin (ENSRNOT00000023412.4) and adrenocorticotropic hormone (ENSRNOT00000016976.7). (C) The number of significantly regulated genes in pituitary from stressed versus control rats (105 up, 212 down, q ≤ 0.05). (D) Top 5 upregulated and top 5 downregulated transcripts, scored by fold-change (b-value), mean ± SEM. (E) Top 20 differentially expressed transcripts, scored by significance (q-value). Data are related to Data S1.

We have recently shown that social stress provoked by the resident-intruder stress paradigm in outbred Sprague-Dawley rats (Figure 1A) is associated with submissive behavior or freeze, reduced weight gain, prolonged hyper-locomotion, and persistent increase of IL-6 mRNA in lymphoid tissues (Jacobsen et al., 2020; Rajalingam et al., 2020). Here, we expand the analyses of these animals to examine the pituitary effects of social stress.

RNA sequencing of pituitary tissues from Sprague-Dawley rats exposed to repeated social defeat versus controls was performed. All samples were included in a principal component analysis (PCA) and—as expected for outbred animals—the plot demonstrated a spread of the individual gene expression, and gene expression overlap in stress conditioned vs. control rats (Figure 1B, left). The main drivers of variance in the PCA were the expression of the pituitary hormones; growth hormone, prolactin, and adrenocorticotropic hormone (Figure 1B, right).

However, by comparing gene expression in stress conditioned vs. control rats, we detected 300 differentially expressed transcripts (DETs) (q ≤ 0.05), where around two-third of the DETs were downregulated (Figure 1C). Ranked by fold-change (b-value), the top 5 upregulated DETs (all protein coding) were tRNA splicing endonuclease subunit 34 (*Tsen34*), ankyrin 3 (*Ank3*), testis expressed 21 (*Tex21*), protein tyrosine phosphatase, receptor type, R (*Ptprr*), and bridge-like lipid transfer protein family member 1 (*Bltp1*). Ranked by fold-change (b-value), the top 5 downregulated DETs (all protein coding) were actin-related protein ⅔ complex, subunit 2 (*Arpc2*), neuronal cell adhesion molecule (*Nrcam*), NPL4 homolog, ubiquitin recognition factor (*Nploc4*), 3′(2),-5′-bisphosphate nucleotidase 2 (*Bpnt2*), and ribophorin II (*Rpn2*) (Figure 1D).

According to NCBI genes summary for human and/or rat, *Tsen34* enables removal of introns from precursor tRNAs; *Ank3* enables several functions like cadherin-binding activity, spectrin-binding activity, and transmembrane transporter-binding activity; *Tex21* is not well described; *Ptprr* is predicted to enable activity of protein kinases and tyrosine phosphatases; and *Bltp1* is predicted to be involved in endocytic recycling. Furthermore, *Arpc2* enables binding activity of several complexes such as ATPase; *Nrcam* enables, among other things, ankyrin binding activity; *Nploc4* enables binding activity of ATPase, ubiquitin, and ubiquitin protein ligase; *Bpnt2* works in humans as a catalyzer in hydrolysis of phosphoadenonsine phosphate to adenosine monophosphate; and *Rpn2* enables ribosome-binding activity.

The top 20 DETs ranked by significance level (q-value) are shown in Figure 1E. All transcripts in this list were protein coding. Notably, *Arpc2*, *Nrcam*, and *Bpnt2* were top rated both by fold-change and by significance level. List of all DETs can be found in Data S1.

### Social stress-induced genes are involved in neuron morphogenesis and communication

**Figure 2:**
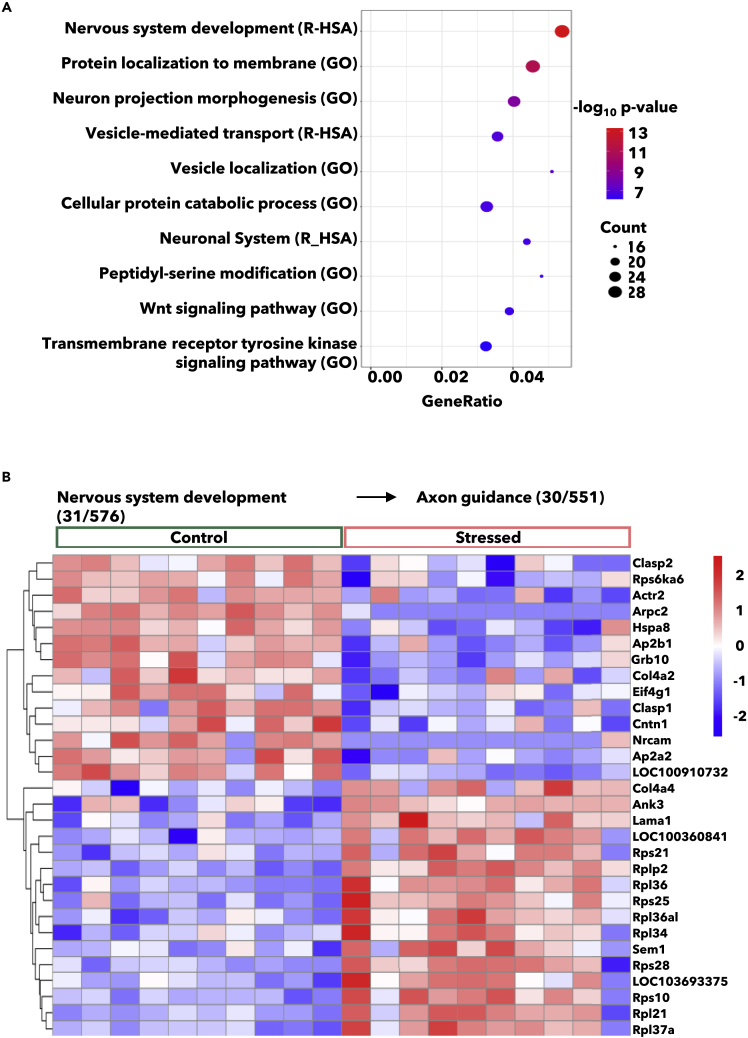
Stress regulated genes are involved in processes related to neuron morphogenesis and communication (A) Top ten significantly enriched pathways and gene ontology terms by gene enrichment analysis. Analysis includes the coding genes of differentially expressed transcripts (DETs) q ≤ 0.05. (B) 30 of 31 DETs linked to the Reactome pathway *Nervous system development* were further linked to the downstream pathway axon guidance. The expression of these 30 DETs is illustrated as a heatmap. Data are related to Figue S1 and Data S1.

To further explore the biological role of the DETs induced by repeated social defeat, we performed gene enrichment analysis (Data S1; RNA-seq Metascape). The top 10 significantly enriched pathways and gene ontology terms are shown (Figure 2A).

The Reactome pathway “Nervous system development” topped the list, followed by the Gene Ontology terms “Protein localization to membrane” and “Neuron projection morphogenesis”. This pathway and these terms include processes involved in neuron morphogenesis and neuron communication, thus the DETs in these processes could be important for translating the pituitary molecular stress response. By further dissecting the Reactome pathway “Nervous system development”, we found that most DETs (30/31) could be mapped to the pathway “Axon Guidance”, which describes the process by which neurons send out axons to reach their synaptic target (Zang et al., 2021). As shown in the heatmap, the majority of the *upregulated* DETs in this pathway code for ribosomal proteins (Figure 2B). Within the axon guidance pathway, these proteins are involved in signaling by ROBO-receptors (Figure S1). Majority of the *downregulated* DETs are in the axon guidance pathway, i.e., involved in L1cam interactions, Eph-ephrin, and Ret signaling (Figue S1). Among the DETs involved in axon guidance, *Ank3*, *Arpc2*, *and Nrcam* and ribosomal protein R36 *(Rpl36)* are also listed as top regulated DETs (Figures 1C, 1D and S2B).

### Social stress induces pituitary epigenetic changes

Previous data show that social stress may influence HPA function in the rat through epigenetic programming (McGowan et al., 2009). Thus, to depict an initial epigenetic pituitary stress response, we performed DNA bisulfite sequencing, mapping genomic levels of 5-methylcyosine (5mC). Samples from five rats with a robust stress response (low weight increase) and five even numbered controls were included for this analysis.

**Figure 3:**
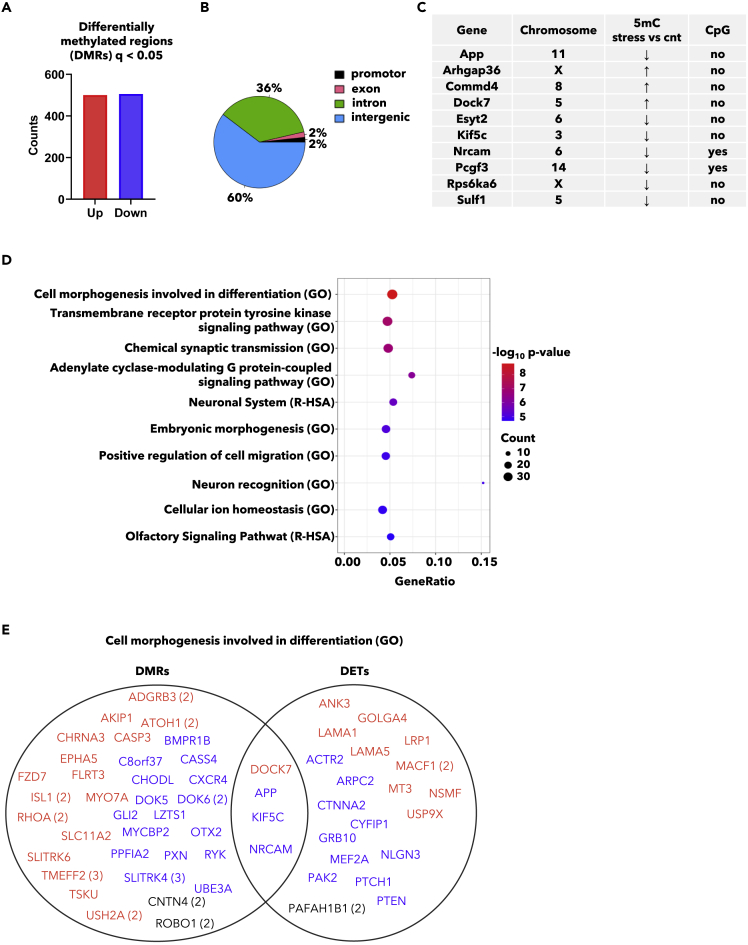
Social stress induces pituitary epigenetic changes (A) Genomic mapping of 5-methylcytosine (5mC) by bisulfite sequencing detected 1005 differently methylated regions (DMRs) in pituitary tissue of stressed (n = 5) vs control rats (n = 5). (B) Circle diagram locating the differently methylated regions according to genomic function. (C) List of genes found to be significantly regulated both by RNA sequencing and by bisulfite DNA sequencing (q < 0.05). (D) Gene enrichment analysis on differently methylated regions based on the closest located gene. Top 10 pathways and gene ontology terms are listed. (E) DMRs and differentially expressed transcripts (DETs) linked to the GO pathway *Cell morphogenesis involved in differentiation* (GO 0000904). Data are related to Data S1 and S2.

Given a significance level of q < 0.01, we found 1005 differentially methylated regions (DMRs), (Figure 3A, Data S1; 5mC). Most of the DMRs (60%) were intergenically located, while the remaining DMRs were mapped to introns (36%), exons (2%), and promoter regions (2%) (Figure 3B). Furthermore, gene enrichment analysis (DMRs were linked to their closest located gene) was performed. Despite a low level of *in*-gene-located DMRs, and low numbers of genes both showing altered methylation status and transcription levels (Figure 3C), the significantly enriched gene ontology terms and pathways well resembled the RNA gene enrichment analysis, involving processes related to neuron morphogenesis and communication (Figure 3D). In line with this, the top three ranked terms “Cell morphogenesis in differentiation”, “transmembrane receptor protein tyrosine kinase signaling pathway”, and “chemical synaptic transmission” were indeed also significantly enriched in the gene enrichment analysis including DETs (Data S1; Metascape comparisons). Comparing the DETs and the DMRs enriched in the top ranked “Cell morphogenesis involved in differentiation”, genes showed both altered methylation status and altered transcription levels in response to stress; amyloid beta precursor protein (*App)*, dedicator of cytokinesis 7 (*Dock7)*, kinesin family member 5C (*Kif5c)*, and *Nrcam* (Figure 3E). According to NCBI genes summary, *App* encodes a cell surface receptor and transmembrane precursor protein that is cleaved by secretases to form a number of peptides. *Dock7* encodes a guanine nucleotide exchange factor that plays a role in axon formation and neuronal polarization. *Kif5C* is a kinesin heavy chain subunit involved in the transport of cargo within the CNS.

Throughout all analyses performed, *Nrcam* was ubiquitous. *Nrcam* was among the top regulated transcripts (Figures 1D and 1E), a part of the most significantly enriched gene ontology terms and pathways (Figure 2B) and contained an CpG-rich area with significantly reduced levels of 5mC after stress (Figure 3C). We therefore continued our study by investigating the significance of *NRCAM* in psychosocial stress in humans.

### A genetic variant of *NRCAM* associates with altered stress response in male humans

**Figure 4:**
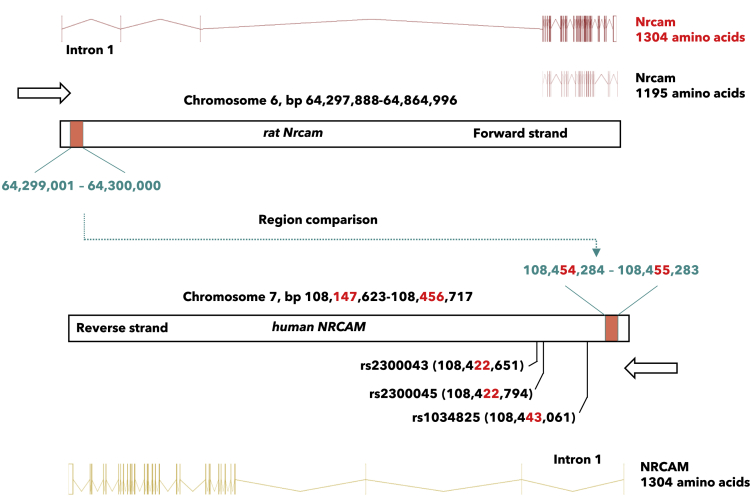
The stress-induced demethylated region of rat *Nrcam* aligns with a regulatory region of human *NRCAM* Illustration showing the two detected *Nrcam* transcripts, *Nrcam*-206 and *Nrcam*-208 (adapted from Ensembl genome browser, version 103: Feb 2021 (Cunningham et al., 2022)). The significantly demethylated region of rat *Nrcam* is located in the gene region corresponding to intron 1 (64,299,001-64,300,000). This region corresponds to a regulatory promoter region of *NRCAM* in humans (108,454,284-108,455,283), located in intron 1. Located in intron 1 is also the human haplotype rs2300043, rs2300045, and rs1034825.

The rat *Nrcam* gene is, in *Ensembl (*version 103: Feb 2021), listed by 14 different transcript variants. As illustrated in Figure 4, we found two regulated *Nrcam* transcripts in our dataset, *one* coding for a 1195 amino acid peptide (transcript-1195) (q = 0.121, b-value = −0.245) and *one* coding for a 1304 amino acid peptide (transcript-1304) (q < 0.0001, b-value = −4.1). The region showing stress-induced downregulation of 5mC was located at intron 1 of the transcript-1304 (chromosome 6, base pairs 64,299,001-64300000). By Ensembl region comparison (Cunningham et al., 2022), we found that this region corresponded to a promoter area in intron 1 in the human *NRCAM* gene (chromosome 7, base pair 108454284-108455283), also coding for intron 1 of five *NRCAM* transcripts including a transcript coding for a 1304 amino acid peptide. Based on the location of the observed methylation changes in the animal experiment, we addressed the role of the equivalent regulatory DNA region in humans just in front of (below) base pair 108454284. This area has previously been linked to regulation of gene expression (Ishiguro et al., 2006). Hence, the earlier described three-nucleotide polymorphism haplotype, i.e., base pair location 108422651-108422794-108443061, characterizing this region in intron 1 (Marui et al., 2009), (Figure 4) was therefore chosen for further analyses.

*NRCAM* genotyping of a cohort consisting of 1205 employees aged 18–60 years, randomly drawn from The Norwegian Central Employee Register, was performed. These employees filled out a questionnaire to measure their experienced levels of workplace bullying and harassment, here in the form of abusive supervision and negative affect. To test the relevance of our *Nrcam* finding from the rodent model, we chose to examine the influence of social stress and genetic variability of the human *NRCAM* gene (intron 1) on negative affect as a parallel to the stress-induced behavioral changes seen in the rats.

**Figure 5:**
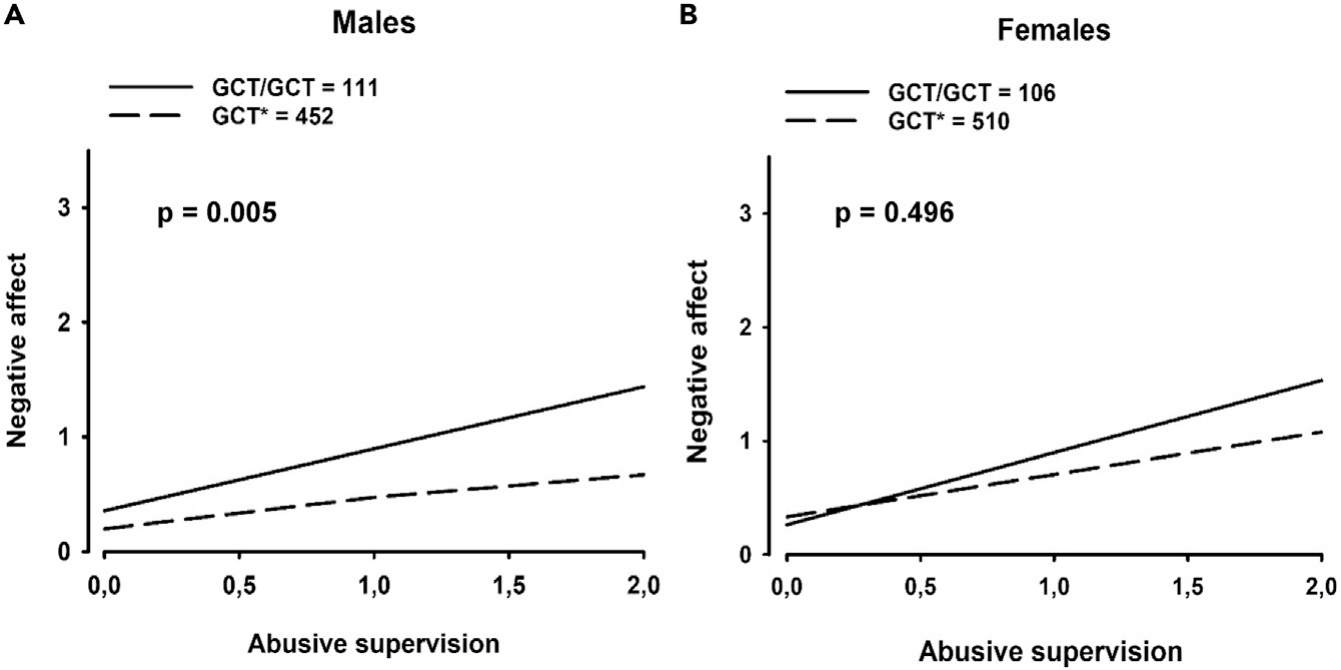
Genetic variant of *NRCAM* associates with negative affect in men exposed to abusive supervision (A) Negative affect in male subjects with *NRCAM* haplotype GCT/GCT versus GCT∗ exposed to abusive supervision (∗ = all other haplotype combinations). (B) Negative affect in female subjects with *NRCAM* haplotype GCT/GCT versus GCT∗ exposed to abusive supervision (∗ = all other haplotype combinations). Related to Table 1, Table 2, Table 3, 2, and 3, and Tables S1–S3.

Based on the frequency of alleles (Table 1 and Table 2, Tables S1 and S2), we studied the moderating effect of two copies of the rs2300043G - rs2300045C - rs1034825T (GCT) NRCAM haplotype versus other combinations on the association between abusive supervision and negative affect. A significant Abusive supervision × GCT interaction was observed, indicating an increased vulnerability in GCT males, but not in females (Table 3). A significant difference between men and women regarding the effect of the haplotype was also revealed (Table S3). In summary, the results indicated vulnerability toward more pronounced stress-induced negative affect in males carrying two copies of the GCT allele (Figure 5).

**Table 1:** Top 3 alleles *NRCAM* haplotype

Allele	Allele frequency
GCT	1110
CTC	853
GTT	258

**Table 2:** Allele combination based on top 2 alleles

Allele combination	Frequency
CTC/GCT	407
GCT/GCT	229
CTC/CTC	136

**Table 3:** Hierarchical regression analysis of the effect of abusive supervision on negative affect; main effects and two-way interaction (Abusive supervision^x^*NRCAM* haplotype) stratified by gender

	Men				Women			
	Coef	Std. Err.	p value	95% CI	Coef	Std. Err.	p value	95% CI
**Step 1**								
**Main effects**								
Abusive supervision	**0.254**	0.046	**0.000**	(0.163, 0.345)	**0.385**	0.044	**0.000**	(0.297, 0.473)
*NRCAM* haplotype								
GCT∗	**−0.146**	0.048	**0.003**	(-0.242, −0.050)	0.043	0.052	0.407	(-0.059, 0.147)

**Step 2**								
Abusive supervision	**0.540**	0.112	**0.000**	(0.319, 0.760)	**0.525**	0.154	**0.001**	(0.221, 0.829)
*NRCAM* haplotype								
GCT∗	0.263	0.155	0.090	(-0.040, 0.568)	0.222	0.195	0.257	(-0.161, 0.606)

**Two-way interaction**								
GCT∗	**−0.342**	0.122	**0.005**	(-0.584, −0.101)	−0.153	0.161	0.344	(-0.470, 0.164)

Coef: coefficient, Std. Err.: SE CI: confidence interval, reference group: GCT/GCT haplotype, ∗: all other haplotype combinations. Bold numbers marks statistical significant values.

Syntax STATA: Step 1*: by Gender, sort: regress NegativeAffect AbusiveSupervision Haplotype*.

Number of observations = 1184.

## Discussion

In the present study, we showed that social stress provoked by the resident-intruder stress paradigm modulates pituitary gene expression and DNA methylation in rats. Furthermore, we found that many stress-regulated genes were involved in neuron morphogenesis and communication. Throughout all analyses performed, the *Nrcam* gene was ubiquitous and further genetic analyses in the human cohort demonstrated that men carrying two copies of the *NRCAM* combination rs2300043G - rs2300045C - rs1034825T, i.e., the haplotype (GCT), were more vulnerable to social stressors than other men. As mentioned above, previous findings show that social stressors may disturb behavior (McGowan et al., 2009; Pich et al., 1993), reduce weight gain, as well as promote inflammation (McKim et al., 2018; Rajalingam et al., 2020; Wohleb et al., 2014). In the present study we extended these earlier analyses by examining global pituitary RNA expression and epigenetic remodeling through DNA methylation.

Interestingly, about 70% of the inbred mice exposed to the resident-intruder paradigm show social avoidance and physiological changes (and are therefore termed susceptible), whereas 30% do not (termed resilient) (Golden et al., 2011). However, these vulnerable versus resilience profiles may not be that clear-cut in rats. The difference between the data of mice and rats could be related to minor differences in the handling of the animals and the resident-intruder protocol, and a suggestion for standardized protocol has recently been published (Munshi et al., 2022). Anyhow, when we previously examined the SPD rats 24 h after the last stress exposure to discover persistent effects on social behavior or weight gain, we did not observe any “resilient” group based on social avoidance among the stress-exposed rats (Jacobsen et al., 2020; Rajalingam et al., 2021), as might be expected if we had carried out the experiment in inbreed mice (Nasca et al., 2019).

Using the resident-intruder protocol of our lab, we have found that reduced weight gain is the most robust response to stress in SPD rats (Jacobsen et al., 2020; Rajalingam et al., 2020). For this reason, weight gain was used to define the responders in the present methylation analyses. In these analyses, the stress-induced gene transcription and stress-induced genomic 5mC regulation showed only a weak overlap. Hence, our data support previous studies demonstrating relatively low overlap between epigenetic histone regulation and gene expression of the prefrontal cortex in mice exposed to social defeat (Reshetnikov et al., 2021). However, digging deeper into the biological processes and pathways, an increased coherence of our epigenetic and gene expression data was seen. Of top ten enriched gene ontology terms in the analysis including DMRs, five was also significantly enriched when analyzing DETs. Both the DETs and DMRs were mostly related to biological pathways such as neurodevelopment, neuron morphogenesis, and neuron communication.

The direction of pathway regulation, i.e., if a pathway is up- or downregulated, is not given by gene enrichment analyses. Thus, we can by this method only estimate directions based on the expression levels of the individual genes. For the pathway axon guidance, transcripts coding for genes involved in L1cam interactions, RET signaling, and Eph-Ephrin signaling were mainly downregulated in stressed animals, while all ribosomal transcripts linked to Robo receptor signaling were upregulated. Axons contain a pool of messenger RNAs, ribosomes for translation and molecules involved in degradation, and local translation of in growth cones and axons is required for axon guidance (reviewed in (Russell and Bashaw, 2018)). Thus, increased number of ribosomal mRNA could also reflect *less* translation of these transcripts. In line with this, levels of Eif4g1, a translation initiation factor promoting ribosome binding to the mRNA (Prevot et al., 2003), appeared to be decreased in stressed animals. Over all, downregulation of the pathway axon guidance would be in line with previous studies finding decreased number of axons in response to chronic stress (Csabai et al., 2018). Furthermore, decreased expression of genes involved in cell-to-cell communication and neuronal synaptic plasticity has also been observed in social defeat stress-induced depression (Bondar et al., 2018).

Interestingly, many of the genes in these networks have previously been linked to, rodent- and human, stress-related conditions (*Ank3* - (Luoni et al., 2016; Rangaraju et al., 2016), *Cntn1* - (Li et al., 2021)). In the present study, however, we have chosen to emphasize the possible role of NRCAM in neurocognitive function. Previous observations suggest that the NRCAM rs2300043/rs2300045/rs1034825 intron 1 haplotype could be a biomarker for autism (Marui et al., 2009). Moreover, the stress-induced demethylated region of rat *Nrcam* gene, aligned to intron 1 of the human *NRCAM* gene, also pointed to the rs2300043/rs2300045/rs1034825 haplotype (base pair location 108422651-108422794-108443061). When investigating the role of this haplotype, i.e., these SNPs covering the human intron 1, we discovered an association between abusive supervision and negative affect in men moderated by the GCT haplotype. Hence, it seems reasonable to suggest that genetic variance in intron 1 just in front of (below) the hypomethylated area of the *NRCAM* gene may be linked to stress-induced regulation of *NRCAM*.

Previous observations indicate that the *NRCAM* gene also affects drug-induced reward, and that genetic variability of the gene may be associated with expression levels as well as substance abuse vulnerability (Ishiguro et al., 2006). Also, data exist that *NRCAM* impacts the function of ADAM10 in neurite outgrowth, which in turn may be important for neurodegenerative processes (Brummer et al., 2019). Moreover, earlier findings have linked *NRCAM* (data of GWAS) to schizophrenia (Zhang et al., 2015). Adding to this, our observations suggest that social stress (abusive supervision) and the genetic variability in the *NRCAM* (the GCT haplotype) interact, moderating the impact of environmental stress on affect and emotions.

Taken together, the present study suggested that social stress, i.e., repeated social defeat, may influence gene expression including *NRCAM* signaling in the pituitary. Also, our data showed that the association between social stress in the form of abusive supervision and negative affect was stronger in men with the *NRCAM* GCT haplotype. Hence, our study goes well with the notion that the genetic variability in intron 1 of the gene encoding *NRCAM* may influence the mechanisms underlying brain development (Marui et al., 2009) and axon growth (Lustig et al., 1999).

### Limitations of the study

How repeated social defeat affects the pituitary gene expression is not fully enlightened in the present study. For example, the genes that respond to stress and the genes associated with stress sensitivity may be different. Also, the present study does not directly address if hypomethylation close to the NRCAM combination rs2300043G - rs2300045C - rs1034825T haplotype in intron 1 of the NRCAM gene may inhibit expression of the transcript NRCAM-204. Hence, how changes in methylation influence the transcript mechanisms and if this process interacts with genetic variability, i.e., in this case the nearest haploblock, still need further examination.

Moreover, the resident-intruder stress paradigm used in the present study is in its design only applicable to male rats or mice. This underscores the challenge with the fact that few pre-clinical models have been designed to study females (Lopez and Bagot, 2021; Shansky, 2019). Hence, a limitation for the present study is the use of a pre-clinical model designed for males only. Our present finding that the association between abusive supervision and negative affect moderated by the *NRCAM* haplotype was only true for men, emphasizes the problem. We and others should for future studies plan for pre-clinical models validated for both sexes.

Finally, few subjects reported high levels of abusive supervision (9.6% had a score of 0.8 or more). These findings merit further examinations on how high levels of abusive supervision influences negative affect.

In summary, the present data suggest that social stress such as repeated social defeat may modulate pituitary RNA expression and DNA methylation. Our findings also emerge the hypothesis that repeated social defeat may trigger a genetic mechanism characterized by changed pituitary *Nrcam* gene expression. Moreover, the present results show that the association between social stress and negative affect in humans is moderated, i.e., increased, by the NRCAM rs2300043G - rs2300045C - rs1034825T haplotype in intron 1. We conclude that the expression of the *Nrcam* gene may be affected by social stress and that genetic variability in *NRCAM* intron 1 region also influences stress-induced negative emotions.

## STAR★Methods

### Key resources table

**Table:** 

REAGENT or RESOURCE	SOURCE	IDENTIFIER
**Biological samples**		
Pituitary from male Sprague Dawley rats	Provided by Johannes Gjerstad	Approval FOTS-8212
DNA collected from human salvia samples	Provided by Johannes Gjerstad	Approval REK 2014/1725
Allprep DNA/RNA Micro kit	Qiagen	ID80224

**Deposited data**		

ArrayExpress	This study	https://www.ebi.ac.uk/arrayexpress/, by accession number E-MTAB-11285
ArrayExpress	This study	https://www.ebi.ac.uk/arrayexpress/, by accession number E-MTAB-11290
Code for RNA-seq analysis	This study	https://doi.org/10.6084/m9.figshare.17212532
Code for Bisulfite-seq analysis	This study	https://doi.org/10.6084/m9.figshare.17212571

**Experimental models: Organisms/strains**		

Sprague–Dawley rats	Janvier Labs; France	RjHan:SD
Long Evans rats	Envigo; USA	HsdBlu:LE

**Software and algorithms**		

Trimming sequencing raw files (RNA and bisulfite)	fastp (v0.20.0)	Chen et al.,2018
Filtered reads mapped to cDNA	Kallisto (v0.46.2)	Bray et al., 2016
Obtaining DETs	Sleuth (v0.30.0) R package	Pimentel et al., 2017
Gene enrichment analyses	Metascape	Zhou et al., 2019
Gene enrichment analyses and illustrations	Reactome	Gillespie et al., 2022; Jassal et al., 2020
Detecting methylations	Bismark (v0.22.3)	Krueger and Andrews, 2011
DMRs	methylKit (v1.14.2) R package	Akalin et al., 2012
Defining haplotype	Phase v2.1.1	Stephens et al.,2001

### Resource availability

#### Lead contact

Further information and requests for resources and reagents should be directed to and will be fulfilled by the Lead Contact, Dr. Maria Belland Olsen (maribol@medisin.uio.no).

#### Materials availability

This study did not generate new unique reagents.

### Experimental model and subject details

#### Animals

Male Long Evans rats (age; 100-120 days, 500–550 g) housed with a female Long Evans rat (age; 50-56 days, 200–250 g) in a 0.56 m^2^ cage. Male Sprague–Dawley rats (age; 50-56 days, 300–400 g) housed in pairs. The different strains (Long Evans rats from Envigo; USA and Sprague–Dawley rats from Janvier Labs; France) were kept in separate rooms. All rats were acclimatized to a 12:12 h light-dark cycle, ventilation rate of 15 × air per hour, 21–22°C and 45–55% humidity. At all times, the rats had *ad libitum* access to food and water. Bedding was changed once a week. All animal procedures were approved by the Norwegian Food Safety Authority and performed in conformity with laws and regulations controlling experiments and procedures on live animals in Norway (FOTS-8212).

#### Human cohort

The data were based on a probability sample of 5000 employees (from 18 to 60 years) randomly drawn from The Norwegian Central Employee Register collected by Statistics Norway (Jacobsen et al., 2018). Briefly, a total of 1608 persons (32 %) returned the questionnaire (distributed by the Norwegian Postal Service in 2015). Samples with missing data were excluded and a total of 1179 persons (616 females and 563 males) were included in the statistical analysis (Data S3). The survey was approved by the Regional Committee for Medical Research for Eastern Norway (REK 2014/1725).

### Method details

#### Resident-intruder paradigm

The first part of the present study was based on our previous work i.e., previously harvested pituitary tissues from rats exposed to social stress induced by the resident-intruder stress paradigm (Rajalingam et al., 2020). A resident-intruder paradigm, where Sprague–Dawley intruder rats were exposed to social stress by dominant Long Evans resident rats; 1 h each day (between 09:00 and 13:00) for 7 consecutive days, was used.

To ensure dominant behavior of Long Evan males i.e., the resident rats in the paradigm, a screening was performed prior to the stress-conditioning week. Top ten aggressive rats were chosen based on the highest incidences of attacks over a period of 10 min.

First, the female rat was temporarily removed from the resident cage 1 h before the stress conditioning. Next, the stress conditioning was performed by introducing the intruder animal into the resident cage. The male resident and intruder rat were separated upon three episodes of social defeat (submissive supine posture, freeze or flight), or after 10 min of interaction by a perforated plastic wall, allowing the intruder rat to still see, smell and hear the resident rat. Finally, after 60 min in the resident cage, the intruder rat was returned to its home cage, and the female rat was returned to the resident cage. The conditioning procedure described above was repeated for 7 days (day 0-6, Figure 1A). To prevent habituation to the dominance establishment with the resident rat, the intruder animals were introduced to a new resident animal every day. The animals randomized to control followed the same procedure except that they visited a foreign cage without a resident rat.

#### Tissue harvest

24 h after last stress exposure and following social interaction test and 1-h rest in their home cage, the Sprague-Dawley intruder and control rats were euthanized by dislocation of the neck under isoflurane anesthesia. The pituitary gland was harvested, frozen on liquid nitrogen and stored at −80°C.

#### RNA sequencing and processing

To explore the initial pituitary gene expression in 10 stressed rats versus 10 controls, total RNA was isolated from the tissues (Qiagen AllPrep ID80224) and mRNA sequencing (Novogene Co., Ltd, China) were performed. The mRNA sequencing raw files were trimmed with fastp (v0.20.0) (Chen et al., 2018) in paired-end mode to remove adapters and low-quality reads with phred score below 30. Filtered reads were then mapped to rat cDNA (Rnor_6.0, Ensembl release 99) (Howe et al., 2021) using Kallisto (v0.46.2) with 200 bootstrap iterations (Bray et al., 2016).

Sleuth (v0.30.0) R package was used to obtain differentially expressed transcripts (DE-Ts) with Wald test, including beta values being the effect size on the natural-log transformed data, an estimator of the fold change (Pimentel et al., 2017). Gene enrichment analysis were performed by Metascape (Zhou et al., 2019) and Reactome (Gillespie et al., 2022; Jassal et al., 2020), including DE-Ts q ≤ 0.05.

#### Bisulfite (5mC) sequencing and processing

In attempt to depict an initial epigenetic pituitary stress response, we performed DNA isolation (Qiagen Allprep) and bisulfite DNA sequencing (Novogene Co., Ltd, China), mapping pituitary genomic levels of 5mC. The DNA were fragmented into 200-400bp using Covaris S220. Then, the terminal repairing, A-ligation and methylation sequencing adapters ligation were performed with the DNA fragments. The EZ DNA Methylation Gold Kit (Zymo Research, CA) was used to perform the bisulfite treatment, where the unmethylated cytosines were changed into uracil while methylated cytosines remained unchanged. Next, the treated DNA was amplified by PCR and sequenced. To analyze the bisulfite DNA sequencing reads, 15 bases from the front, 10 bases from the tail, poly A/T/C/G tails, adapters and phred score below 30 of raw reads were trimmed off by fastp (v0.20.0) (Chen et al., 2018) in paired-end mode. Filtered reads were mapped to rat genome (Rnor_6.0, Ensembl release 99) (Howe et al., 2021) and methylations were called by Bismark (v0.22.3) (Krueger and Andrews, 2011). DMRs were obtained by methylKit (v1.14.2) R package (Akalin et al., 2012). Samples from 5 rats with a robust stress response (defined by weight gain < median weight gain) and 5 representative controls (even numbered controls) were then compared. DE-Ts for samples used for bisulfite DNA sequencing (5 stressed vs 5 controls) are given in Data S2.

#### NRCAM genotyping

Single nucleotide polymorphism (SNP) genotyping was carried out using predesigned TaqMan SNP genotyping assays (Applied Biosystems, Foster City, CA, USA), for details see also (Jacobsen et al., 2018). In accordance with the procedure in our earlier studies (Jacobsen et al., 2018; Rajalingam et al., 2019), an ABI 79000HT sequence detection system was used. Negative controls were included in every run. Approximately 10% of the samples were re-genotyped and the concordance rate was 100%. Phase v2.1.1 was used to define the *NRCAM* haplotypes (Stephens et al., 2001).

#### Questionnaire

The respondents were asked to indicate if they experienced abusive supervision based on a 5-item version of the Tepper’s 2000 scale. The response categories ranged from 0 to 4 (‘never’, ‘rarely’, ‘once in a while’, ‘quite often’ and ‘very often or always’) (Abdul Hamid et al., 2016; Tepper, 2000). (Cronbach’s alpha for abusive supervision was 0.87.) In addition, the respondents were asked report experienced negative affect measured by the Negative Affect Schedule (I-PANAS-SF) during the last two weeks (Thompson, 2007). The response categories for negative affect ranged from 0 to 4 (‘not bothered,’ ‘a little bothered,’ ‘considerably bothered’, ‘seriously bothered’). Additionally, the respondents reported on their age, sex and their heritage. Heritage was reported by stating if their grandparents were born outside of Europe (yes or no).

### Quantification and statistical analysis

#### Association analyses

For the abusive supervision variable, a mean value was calculated from the 5-items in the questionnaire. Haplotyping was based on the following SNPs; rs2300043, rs2300045 and rs1034825 - corresponding to the region of interest. The haplotype combinations were dichotomised into two copies of the GCT alleles vs all other combinations. Linear regression (stratified by gender, adjusted for age and heritage) was conducted to assess for potential associations between abusive supervision and negative affect. In the analyses, the main effects (without any interaction term) were assessed in step 1, whereas the two-way interactions (interaction term; abusive supervision x haplotype) were provided in step 2. To assess for gender differences, a linear regression including a three-way interaction (abusive supervision x haplotype x gender) was conducted (see Table S3).

A Chi square test calculator (available from http://quantpsy.org) was used to assess deviation from the Hardy-Weinberg equilibrium. No deviation from the Hardy-Weinberg equilibrium for any of the SNPs. All other statistical analyses were conducted using Stata SE 16.0 (StataCorp. 2019. Stata Statistical Software: Release 16. College Station, TX: StataCorp LLC). Significance was accepted at the p < 0.05 level.

## Data Availability

• Bulk RNA-seq and bisulfite-seq data are available at ArrayExpress. Accession numbers are listed in the key resources table. • All original code has been deposited at figshare and DOIs are listed in the key resources table. • Any addition information required to reanalyse the data reported in this paper is available from the lead contact upon request

## Supplemental information

Document S1. Figure S1 and Table S1–S3

Data S1. seq_RNA_bisulfite, related to Figures 1–3 DE-Ts and DMRs for 10 stressed vs. 10 control rats.

Data S2. seq_RNA_5vs5, related to Figure 3 Comparison of RNA expression (DE-Ts) for 5 stressed vs. 5 control rats corresponding to the (5 + 5) samples used for bisulfite sequencing.

Data S3. Human cohort, related to Figure 5 Human cohort, raw data.

## References

[bib1] Abdul Hamid R., Juhdi N., Ismail M., Abdullah N.A., Hamid A. (2016). Abusive supervision and workplace deviance as moderated by spiritual intelligence: an empirical study of Selangor employees. Mal. J. Society Space.

[bib2] Akalin A., Kormaksson M., Li S., Garrett-Bakelman F.E., Figueroa M.E., Melnick A., Mason C.E. (2012). methylKit: a comprehensive R package for the analysis of genome-wide DNA methylation profiles. Genome Biol..

[bib3] Bagot R.C., Parise E.M., Pena C.J., Zhang H.X., Maze I., Chaudhury D., Persaud B., Cachope R., Bolanos-Guzman C.A., Cheer J.F. (2015). Ventral hippocampal afferents to the nucleus accumbens regulate susceptibility to depression. Nat. Commun..

[bib4] Bondar N., Bryzgalov L., Ershov N., Gusev F., Reshetnikov V., Avgustinovich D., Tenditnik M., Rogaev E., Merkulova T. (2018). Molecular adaptations to social defeat stress and induced depression in mice. Mol. Neurobiol..

[bib5] Bray N.L., Pimentel H., Melsted P., Pachter L. (2016). Near-optimal probabilistic RNA-seq quantification. Nat. Biotechnol..

[bib6] Brummer T., Müller S.A., Pan-Montojo F., Yoshida F., Fellgiebel A., Tomita T., Endres K., Lichtenthaler S.F. (2019). NrCAM is a marker for substrate-selective activation of ADAM10 in Alzheimer's disease. EMBO Mol. Med..

[bib7] Chen S., Zhou Y., Chen Y., Gu J. (2018). fastp: an ultra-fast all-in-one FASTQ preprocessor. Bioinformatics.

[bib8] Csabai D., Wiborg O., Czeh B. (2018). Reduced synapse and axon numbers in the prefrontal cortex of rats subjected to a chronic stress model for depression. Front. Cell. Neurosci..

[bib9] Cunningham F., Allen J.E., Allen J., Alvarez-Jarreta J., Amode M.R., Armean I.M., Austine-Orimoloye O., Azov A.G., Barnes I., Bennett R. (2022). Ensembl 2022. Nucleic Acids Res..

[bib10] Gillespie M., Jassal B., Stephan R., Milacic M., Rothfels K., Senff-Ribeiro A., Griss J., Sevilla C., Matthews L., Gong C. (2022). The reactome pathway knowledgebase 2022. Nucleic Acids Res..

[bib11] Glambek M., Nielsen M.B., Gjerstad J., Einarsen S. (2018). Gender differences in the relationship between workplace bullying and subjective back and neck pain: a two-wave study in a Norwegian probability sample. J. Psychosom. Res..

[bib12] Golden S.A., Covington H.E., Berton O., Russo S.J. (2011). A standardized protocol for repeated social defeat stress in mice. Nat. Protoc..

[bib13] Harper D.E., Ichesco E., Schrepf A., Hampson J.P., Clauw D.J., Schmidt-Wilcke T., Harris R.E., Harte S.E. (2018). Resting functional connectivity of the periaqueductal gray is associated with normal inhibition and pathological facilitation in conditioned pain modulation. J. Pain.

[bib14] Herman J.P., Ostrander M.M., Mueller N.K., Figueiredo H. (2005). Limbic system mechanisms of stress regulation: hypothalamo-pituitary-adrenocortical axis. Prog. Neuro-Psychopharmacol. Biol. Psychiatry.

[bib15] Hing B., Braun P., Cordner Z.A., Ewald E.R., Moody L., McKane M., Willour V.L., Tamashiro K.L., Potash J.B. (2018). Chronic social stress induces DNA methylation changes at an evolutionary conserved intergenic region in chromosome X. Epigenetics.

[bib16] Howe K.L., Achuthan P., Allen J., Allen J., Alvarez-Jarreta J., Amode M.R., Armean I.M., Azov A.G., Bennett R., Bhai J. (2021). Ensembl 2021. Nucleic Acids Res..

[bib17] Huang J., Guo G., Tang D., Liu T., Tan L. (2019). An eye for an eye? Third parties' silence reactions to peer abusive supervision: the mediating role of workplace anxiety, and the moderating role of core self-evaluation. Int J. Environ. Res. Public Health.

[bib18] Ishiguro H., Liu Q.R., Gong J.P., Hall F.S., Ujike H., Morales M., Sakurai T., Grumet M., Uhl G.R. (2006). NrCAM in addiction vulnerability: positional cloning, drug-regulation, haplotype-specific expression, and altered drug reward in knockout mice. Neuropsychopharmacology.

[bib19] Jacobsen D.P., Eriksen M.B., Rajalingam D., Nymoen I., Nielsen M.B., Einarsen S., Gjerstad J. (2020). Exposure to workplace bullying, microRNAs and pain; evidence of a moderating effect of miR-30c rs928508 and miR-223 rs3848900. Stress.

[bib20] Jacobsen D.P., Nielsen M.B., Einarsen S., Gjerstad J. (2018). Negative social acts and pain: evidence of a workplace bullying and 5-HTT genotype interaction. Scand. J. Work. Environ. Health.

[bib21] Jassal B., Matthews L., Viteri G., Gong C., Lorente P., Fabregat A., Sidiropoulos K., Cook J., Gillespie M., Haw R. (2020). The reactome pathway knowledgebase. Nucleic Acids Res..

[bib22] Krueger F., Andrews S.R. (2011). Bismark: a flexible aligner and methylation caller for Bisulfite-Seq applications. Bioinformatics.

[bib23] Li S., Cao W., Zhou S., Ma M., Zhang W., Li F., Li C. (2021). Expression of Cntn1 is regulated by stress and associated with anxiety and depression phenotypes. Brain Behav. Immun..

[bib24] Lopez J., Bagot R.C. (2021). Defining valid chronic stress models for depression with female rodents. Biol Psychiatry.

[bib25] Luoni A., Massart R., Nieratschker V., Nemoda Z., Blasi G., Gilles M., Witt S.H., Suderman M.J., Suomi S.J., Porcelli A. (2016). Ankyrin-3 as a molecular marker of early-life stress and vulnerability to psychiatric disorders. Transl. Psychiatry.

[bib26] Lustig M., Sakurai T., Grumet M. (1999). Nr-CAM promotes neurite outgrowth from peripheral ganglia by a mechanism involving axonin-1 as a neuronal receptor. Dev. Biol..

[bib27] Mancini G.F., Marchetta E., Pignani I., Trezza V., Campolongo P. (2021). Social defeat stress during early adolescence confers resilience against a single episode of prolonged stress in adult rats. Cells.

[bib28] Marui T., Funatogawa I., Koishi S., Yamamoto K., Matsumoto H., Hashimoto O., Nanba E., Nishida H., Sugiyama T., Kasai K. (2009). Association of the neuronal cell adhesion molecule (NRCAM) gene variants with autism. Int. J. Neuropsychopharmacol..

[bib29] McGowan P.O., Sasaki A., D'Alessio A.C., Dymov S., Labonté B., Szyf M., Turecki G., Meaney M.J. (2009). Epigenetic regulation of the glucocorticoid receptor in human brain associates with childhood abuse. Nat. Neurosci..

[bib30] McKim D.B., Weber M.D., Niraula A., Sawicki C.M., Liu X., Jarrett B.L., Ramirez-Chan K., Wang Y., Roeth R.M., Sucaldito A.D. (2018). Microglial recruitment of IL-1β-producing monocytes to brain endothelium causes stress-induced anxiety. Mol. Psychiatr..

[bib31] Mitchell M.S., Ambrose M.L. (2007). Abusive supervision and workplace deviance and the moderating effects of negative reciprocity beliefs. J. Appl. Psychol..

[bib32] Munshi S., Ritger A., Rosenkranz A.J. (2022). Induction of repeated social defeat stress in rats. Bio. Protoc..

[bib33] Nasca C., Menard C., Hodes G., Bigio B., Pena C., Lorsch Z., Zelli D., Ferris A., Kana V., Purushothaman I. (2019). Multidimensional predictors of susceptibility and resilience to social defeat stress. Biol Psychiatry.

[bib34] Nielsen M.B., Gjerstad J., Jacobsen D.P., Einarsen S.V. (2017). Does ability to defend moderate the association between exposure to bullying and symptoms of anxiety?. Front. Psychol..

[bib35] Pich E.M., Heinrichs S.C., Rivier C., Miczek K.A., Fisher D.A., Koob G.F. (1993). Blockade of pituitary-adrenal axis activation induced by peripheral immunoneutralization of corticotropin-releasing factor does not affect the behavioral response to social defeat stress in rats. Psychoneuroendocrinology.

[bib36] Pimentel H., Bray N.L., Puente S., Melsted P., Pachter L. (2017). Differential analysis of RNA-seq incorporating quantification uncertainty. Nat. Methods.

[bib37] Pizarro J.M., Lumley L.A., Medina W., Robison C.L., Chang W.E., Alagappan A., Bah M.J., Dawood M.Y., Shah J.D., Mark B. (2004). Acute social defeat reduces neurotrophin expression in brain cortical and subcortical areas in mice. Brain Res..

[bib38] Prevot D., Darlix J.L., Ohlmann T. (2003). Conducting the initiation of protein synthesis: the role of eIF4G. Biol. Cell.

[bib39] Rajalingam D., Jacobsen D.P., Nielsen M.B., Einarsen S.V., Gjerstad J. (2019). Exposure to workplace bullying, distress, and insomnia: the moderating role of the miR-146a genotype. Front. Psychol..

[bib40] Rajalingam D., Nymoen I., Jacobsen D.P., Eriksen M.B., Dissen E., Nielsen M.B., Einarsen S.V., Gjerstad J. (2020). Repeated social defeat promotes persistent inflammatory changes in splenic myeloid cells; decreased expression of β-arrestin-2 (ARRB2) and increased expression of interleukin-6 (IL-6). BMC Neurosci..

[bib41] Rajalingam D., Nymoen I., Nyberg H., Nielsen M.B., Einarsen S.V., Gjerstad J. (2021). Workplace bullying increases the risk of anxiety through a stress-induced β2-adrenergic receptor mechanism: a multisource study employing an animal model, cell culture experiments and human data. Int. Arch. Occup. Environ. Health.

[bib42] Rangaraju S., Levey D.F., Nho K., Jain N., Andrews K.D., Le-Niculescu H., Salomon D.R., Saykin A.J., Petrascheck M., Niculescu A.B. (2016). Mood, stress and longevity: convergence on ANK3. Mol. Psychiatry.

[bib43] Reshetnikov V.V., Kisaretova P.E., Ershov N.I., Merkulova T.I., Bondar N.P. (2021). Social defeat stress in adult mice causes alterations in gene expression, alternative splicing, and the epigenetic landscape of H3K4me3 in the prefrontal cortex: an impact of early-life stress. Prog. Neuro-Psychopharmacol. Biol. Psychiatry.

[bib44] Russell S.A., Bashaw G.J. (2018). Axon guidance pathways and the control of gene expression. Dev. Dyn..

[bib45] Sannes A.-C., Christensen J.O., Nielsen M.B., Gjerstad J. (2021). The association between abusive supervision and anxiety in female employees is stronger in carriers of the CRHR1 TAT haplotype. Curr. Res. Behav. Sci..

[bib46] Shansky R.M. (2019). Are hormones a "female problem" for animal research?. Science.

[bib47] Smith S.M., Vale W.W. (2006). The role of the hypothalamic-pituitary-adrenal axis in neuroendocrine responses to stress. Dialogues Clin. Neurosci..

[bib48] Stephens M., Smith N.J., Donnelly P. (2001). A new statistical method for haplotype reconstruction from population data. Am. J. Hum. Genet..

[bib49] Tepper B. (2000). Conseq. Abusive Supervis..

[bib50] Tepper B.J., Simon L., Park H.M. (2017). Abusive supervision. Ann. Rev. Organ. Psychol. Organ. Behavior.

[bib51] Thompson E.R. (2007). Development and validation of an internationally reliable short-form of the positive and negative affect schedule (Panas). J. Cross Cult. Psychol..

[bib52] Vie T.L., Glasø L., Einarsen S. (2012). How does it feel? Workplace bullying, emotions and musculoskeletal complaints. Scand. J. Psychol..

[bib53] Wohleb E.S., McKim D.B., Shea D.T., Powell N.D., Tarr A.J., Sheridan J.F., Godbout J.P. (2014). Re-establishment of anxiety in stress-sensitized mice is caused by monocyte trafficking from the spleen to the brain. Biol Psychiatry.

[bib54] Zang Y., Chaudhari K., Bashaw G.J. (2021). New insights into the molecular mechanisms of axon guidance receptor regulation and signaling. Curr. Top. Dev. Biol..

[bib55] Zhang T.Y., Labonte B., Wen X.L., Turecki G., Meaney M.J. (2013). Epigenetic mechanisms for the early environmental regulation of hippocampal glucocorticoid receptor gene expression in rodents and humans. Neuropsychopharmacology.

[bib56] Zhang Z., Yu H., Jiang S., Liao J., Lu T., Wang L., Zhang D., Yue W. (2015). Evidence for association of cell adhesion molecules pathway and NLGN1 polymorphisms with Schizophrenia in Chinese han population. PLoS One.

[bib57] Zhou Y., Zhou B., Pache L., Chang M., Khodabakhshi A.H., Tanaseichuk O., Benner C., Chanda S.K. (2019). Metascape provides a biologist-oriented resource for the analysis of systems-level datasets. Nat. Commun..

