# Supplementary material for: Mapping of pituitary stress-induced gene regulation connects Nrcam to negative emotions
Source: iScience. 2022 Aug 17;25(9):104953. doi: 10.1016/j.isci.2022.104953 (PMC9437855; doi:10.1016/j.isci.2022.104953)
Supplement: Document S1. Figure S1 and Table S1–S3 [file mmc1.pdf]

iScience, Volume *25*

Supplemental information

Mapping of pituitary stress-induced gene regulation connects Nrcam to negative emotions

Maria Belland Olsen, Ann-Christin Sannes, Kuan Yang, Morten Birkeland Nielsen, Ståle Valvatne Einarsen, Jan Olav Christensen, Ståle Pallesen, Magnar Bjørås, and Johannes Gjerstad

Supplementary Figures

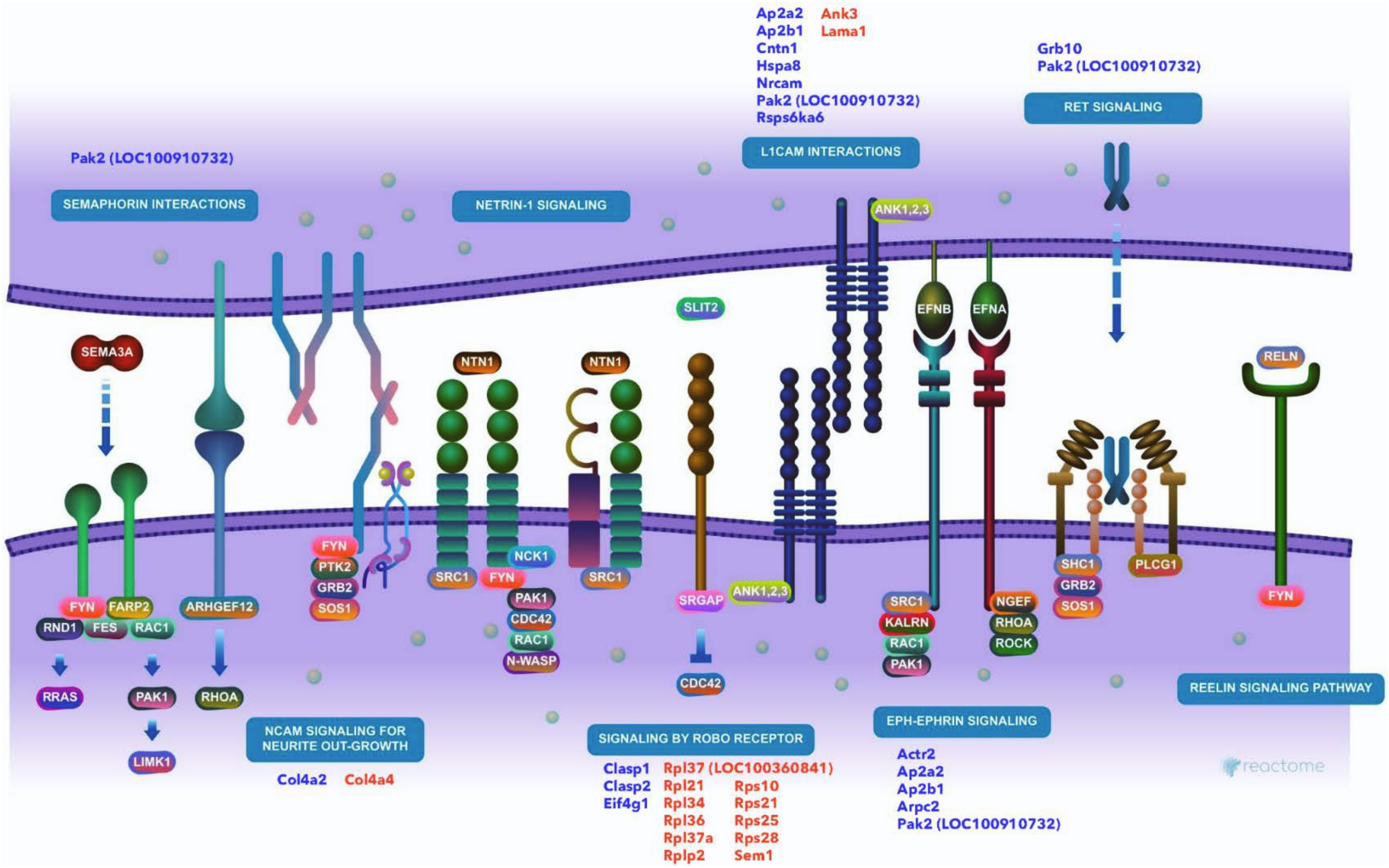

Figure S1. RNA transcripts regulated by stress involved in the Reactome pathway Axon guidance

Illustrated are the differentially expressed transcripts (DETs) (stressed vs. control rats) mapped to the Reactome pathway Axon guidance (R-HAS-422475) (Gillespie *et al.*, 2022). Upregulated DETs marked in red, downregulated DETs marked in blue. Illustration downloaded from Reactome.com. Related to Figure 2.

Supplementary tables

Supplementary table 1. Characteristics of the male subjects by *NRCAM* haplotype (rs2300043, rs2300045, rs1034825); GCT/GCT and GCT*. Related to Figure 5

	Range	GCT/GCT				GCT*				Sum
		N	%	Mean	SEM	N	%	Mean	SEM	
**Subjects**		111	19.6			455	80.4			566
**Negative affect**	0 to 4			0.46	0.05			0.31	0.02	
**Abusive supervision**	0 to 4			0.19	0.03			0.18	0.02	

N: number of subjects, SEM: standard error of mean, *: all other haplotype combinations

Supplementary table 2. Characteristics of the female subjects by *NRCAM* haplotype (rs2300043, rs2300045, rs1034825); TCG/TCG and TCG*. Related to Figure 5

	Range	GCT/GCT				GCT*				Sum
		N	%	Mean	SEM	N	%	Mean	SEM	
**Subjects**		106	17.2			512	82.8			618
**Negative affect**	0 to 4			0.34	0.04			0.40	0.02	
**Abusive supervision**	0 to 4			0.16	0.03			0.19	0.02	

N: number of subjects, SEM: standard error of mean, *: all other haplotype combinations

Supplementary table 3. Linear regression analysis of the effect of abusive supervision on negative affect, three-way interaction (Abusive supervision^{x}Haplotype^{x}Gender). Related to Figure 5

	Coef	Std. Err	p-value	95% CI
**Haplotype - gender difference (reference male)**				
Female (GCT/GCT)	-0.014	0.189	0.941	(-0.386, 0.358)
**Female (GCT*)**	**0.175**	**0.069**	**0.011**	**(0.040, 0.310)**

*Coef: coefficient, Std. Err.: standard error, CI: confidence interval, *: all other haplotype combinations. Bold numbers marks statistical significant values.*

Syntax STATA:
regress NegativeAffect AbusiveSupervision Gender Haplotype Gender#Haplotype c.AbusiveSupervision#Haplotype c.AbusiveSupervision#Haplotype#Gender
